# Reviews

**Published:** 1864-03

**Authors:** 


### REVIEWS.

*A Treatise on Pharmacy. Designed as a Text-Book for the Student, and as a Guide for the Physician and Pharmaceutist, containing the official and unofficial Formulas, and numerous examples of Extemporaneous Prescriptions.* By EDWARD PARRISH, *Graduate in Pharmacy, member of the Philadelphia College of Pharmacy; of the Academy of Natural Sciences of Philadelphia, and of the American Pharmaceutical Association; Principal of the School of Practical Pharmacy, Philadelphia. Third Edition, thoroughly Revised and Improved, with Important Additions, with Two Hundred and Thirty-Eight Illustrations.* Philadelphia, BLANCHARD & LEA, 1864.

This is a complete treatise upon Pharmacy, and contains a great amount of information not connected with official preparations. There are introduced in this edition working formulas for many official preparations, from the new edition of the National Pharmacopœia, which will be found to increase the value of the work, especially to the practical Pharmaceutist. These are followed by remarks upon the properties and uses of these compounds. The process for preparing and dispensing medicines are separately described and illustrated. Chemical compounds are arranged in a manner to show their composition, properties and doses. Formulas are introduced which cannot fail to be of advantage to those who are interested, and the work is made exceedingly valuable by the complete history it contains of the various medical agents, and their uses.

In looking over the remarks under this last head, we are pained to see how easy and natural it is to say of almost all preparations "it is unquestionably a valuable auxiliary to the physician in the treatment of diseases." The above endorsement is placed after preparations as worthless, in our opinion, as Pop Beer, and still they find a place in the works on Pharmacy, and worse still, are represented as valuable in the treatment of what is denominated "Pulmonary Affections," or "Kidney Diseases," or some other equally indefinite and unscientific classification. It is well said however; medicines may be "valuable to the physician," but of no use to patients.

What does the Pharmaceutist mean when he says that "Tar Beer is unquestionably a valuable auxiliary to the physician in the treatment of pulmonary diseases?" Is there any disease of the lung that Tar Beer will cure? We hope the next edition will be definite enough to tell us what pulmonary disease is cured by it. This habit of recommending the various medicinal preparations is not peculiar to the book before us, it is observable in all similar works. Immortal honor is reserved for the author of a work upon Materia Medica which treats only of medicines that are really of any value, and assigns to each its relative and actual importance.

This book commends itself highly to the Physician and Druggist, while the medical student cannot select a more complete work.

---

*Proceedings of the American Pharmaceutical Association at its Eleventh Annual Meeting, held in Baltimore, Md., September,* 1863. *Also the Constitution and Roll of Members. Philadelphia:* Merrihew & Thompson, *Printers,* 1863.

This volume is very creditable to the Association; its reports are attracting attention among scientific men in all countries. The Report upon the Progress of Pharmacy is perhaps the one of most general interest; it deserves attention, and will no doubt occupy a prominent place as a record of observations and discoveries. The Report on the Drug Market is also full of interest, and contains a great amount of information both in a commercial and scientific point of view. We regret not being able to reprint portions of this Report. What is said of the leading articles of the Materia Medica are of great importance to the physician, and will afford the key to some of the disappointments which he meets in the administration of drugs. "Instead of a cinchona containing 2 or 3 per cent. of alkaloid, pharmaceutical preparations of the present day, are made from those which contain the half of one per cent. and cost from 15 to 75 cents per pound."

Eleven specimens of Tinc. Opii of the Pharmacopœia, obtained from the best dealers were examined, and gave the following results; Five were in the neighborhood of seven-twelfths, five vary between eight and ten-twelfths, while one was just about half the proper officinal strength.

This book contains Special Reports and Essays; on Aconite Root, By William Proctor, jr. On Leaves and Seeds of Conium Maculatum, By George C. Close. On Solutions of Tartaric Acid, By John M. Maisch. On Still for Apothecaries, By William Proctor, jr. On Still for Pharmaceutical purposes, By Thomas Wiegand. On Buchu Leaves, By P. W. Bedford. On Preservation of Volatile Oils, By Alfred B. Taylor. On Therapeutical Properties of Sanguinarina, By R. P. Thomas, M. D. On Fluid Extracts, By William Proctor, jr. On the Active Principles of the Strychnaceæ, By Ferdinand F. Mayer. On the Constitution of the Impure Oxides of Iron used in Medicine, By Ferris Bringhurst. On the Contamination of American Sulphuric Acid with Arsenic, By John M. Maisch. On the Extraction of Potassa from Marl, By George J. Scattergood. Also Essays on several other topics, all worthy of most careful perusal.

The following subjects for prizes, open to general competition; the awards to be determined and adjudged by a committee to be appointed at the meeting in 1864, agreeably to a resolution of the Association, adopted August 29, 1862. (See Proceedings for 1862, vol. x, pages 47, 48.)

1—An Essay on Cimicifuga Racemosa in its Chemical and Pharmaceutical Relations and Medicinal Uses.

2—An Essay based on a Practical and Successful Experiment on the Culture and Preparation of Elaterium in the United States, accompanied by a specimen of the product, of not less than 120 grains.

